# Food Access Worries, Food Assistance Use, Purchasing Behavior, and Food Insecurity Among New Yorkers During COVID-19

**DOI:** 10.3389/fnut.2021.647365

**Published:** 2021-08-26

**Authors:** Lauren A. Clay, Stephanie Rogus

**Affiliations:** ^1^Department of Emergency Health Services, University of Maryland Baltimore County, Baltimore, MD, United States; ^2^Department of Family and Consumer Sciences, New Mexico State University, Las Cruces, NM, United States

**Keywords:** COVID-19, food security, food access, food assistance, food purchase behavior

## Abstract

The coronavirus disease of 2019 (COVID-19) disrupted health, economy, and food systems across the United States. This cross-sectional study examined the relationship between food access worries, food assistance use, and purchasing behaviors and food insecurity during COVID-19 among residents of New York State. New Yorkers were recruited to complete a web-based survey through Qualtrics. The survey took place in the summer and fall of 2020 and asked respondents about food access worries, food assistance use, food insecurity, and food purchasing behaviors. Chi-square analysis examined the relationships between food concerns, food assistance use, purchasing behaviors, and demographic characteristics by reported food insecurity, and significant results were analyzed in a series of logistic regression models. Results showed that higher food worries, Supplemental Nutrition Assistance Program (SNAP) use, reported food assistance and delivery as food sources, and self-reported Hispanic ethnicity were associated with a higher likelihood of experiencing food insecurity. Future research is needed to assess the ongoing impacts of the pandemic on food access and food insecurity, particularly among underserved groups. Measures that provide additional money for food and improved food access can alleviate barriers to accessing enough healthy food at this time.

## Introduction

The coronavirus disease of 2019 (COVID-19) had a major impact on health, social life, and the economy across the globe. Over 71 million infections and 1 million deaths have been reported worldwide, with over 22% of cases and 29% of deaths occurring in the United States (1). Social distancing measures were implemented in order to contain the spread of the disease, which led to employment and food system disruptions. These disruptions resulted in business closures that forced about 22 million Americans into unemployment in April 2020 and increased rates of food insecurity, which includes the ability to consistently acquire nutrient-rich, desirable, and varied foods (2–7).

Increases in the volume of food purchased by consumers placed a strain on an inflexible food supply chain that failed to respond to the pandemic (8). Illness among workers in processing facilities due to shared housing and transportation and close proximity of employees in work environments reduced facility capacity (9, 10). This led to many farmers disposing of foods that required further processing (11). Further, food shortages in stores, concerns about food safety (12–14), and localized stay-at-home orders changed purchasing behaviors of consumers. Consumers increased their use of online ordering for pick-up and delivery from restaurants and grocery stores, increased their time spent cooking at home, and reduced overall purchases from restaurants (15). At the same time, food supply and work disruptions widened existing disparities in food access (6). Black and Hispanic Americans are more likely to work essential jobs, such as those in retailing, transportation, manufacturing, healthcare, construction, and the food system, which pay lower wages and lack flexibility and paid sick leave (16–21). These jobs were more likely lost during the pandemic, which further exacerbated financial issues among groups that were already more likely to suffer from food insecurity prior to the pandemic (22, 23).

Several studies have examined food access issues during COVID-19, mostly within the context of food insecurity. Studies have reported increased food insecurity since COVID-19 and issues with food access among food-insecure populations (4, 5). Those experiencing food insecurity reported higher levels of worry about food access and challenges related to food access, including the ability to find the types of foods desired, getting enough food through food assistance programs and emergency food organizations, and an inability to afford to stock up on food for 2 weeks as recommended (4, 5). Food-insecure individuals were also less likely to have sick and vacations days and more likely to report that they would lose their job if they missed too much work (4). This study extends the current research area by examining the relationship between food purchasing behaviors and food insecurity.

In March 2020, when COVID-19 was declared a pandemic by the WHO (24), New York became the epicenter of the virus in the United States (25). On March 20, 2020, Governor Cuomo issued the Executive Order New York State on Pause, a stay-at-home order and closure of all non-essential businesses in the state to slow the spread of the virus (26). As of December 2020, there were over 28,000 deaths from COVID-19 in NY State. Deaths disproportionately affected racial and ethnic minorities. Hispanic New Yorkers accounted for 34% of the deaths but only 29% of the state population and Black New Yorkers accounted for 28% of the deaths but only 22% of the population (27). The purpose of this study was to assess the relationship between food access concerns, food assistance use, and purchasing behaviors and food insecurity in a sample that oversampled New York State residents who were Black, Hispanic, and low-income or low-education during COVID-19.

## Materials and Methods

### Study Design and Sample

A cross-sectional proportional quota sample of 525 New Yorkers was recruited by Qualtrics from their survey panels to complete a web survey. The survey was adapted from a validated food access survey developed by the National Food Access and COVID-19 Research Team (NFACT) (28–30). It included open- and closed-ended questions about food sources, food security, purchasing behavior, food assistance, risk factors for COVID-19, and social determinants of health. Quotas were set to recruit 50% Black or African American, 50% Hispanic, and 50% low-income or low-education participants to oversample groups with an increased risk for food insecurity and for adverse consequences related to COVID-19 (19, 20, 31, 32). Individual panel members were classified as eligible to participate if they were age 18 or older and resided in New York State, excluding New York City. Potential study participants were asked about their race, ethnicity, income, education, and age to evaluate fit with the quotas. Potential participants that met the inclusion criteria but fell outside of the quotas needed to fill the sample were classified as ineligible, and the survey was ended. A response rate is not available with quota data collection through Qualtrics panels because the number of people invited to participate in the survey is not reported by Qualtrics.

### Data Collection

Data from Black or African American and Hispanic participants were collected from July 15 to September 18, 2020, and data from non-Hispanic white participants were collected from December 7–11, 2020. Data on non-Hispanic white participants were collected approximately 3 months after data on Black and Hispanic participants. Case counts were higher during data collection from non-Hispanic white participants; however during both times of data collection, COVID-19 restrictions policies were similar with a regional and clustered approach based on positive rates and hospital capacity and when looking at county level data; during both periods of data collection, there was a significant variation in COVID case counts (33, 34). The median time to complete the survey was 16 min. To ensure quality responses, five quality checks were included within the survey to check for agreement between responses that should have been consistent, for example, an agreement between repeated questions about demographic characteristics. Respondents with a flag for more than two of the quality checks were excluded from the final sample. Surveys completed in faster than half the median time were replaced due to low quality. Finally, survey responses were evaluated to identify poor-quality responses and removed if there was evidence of straight-lining, gibberish, and non-sense answers in accordance with recommended quality review criteria by Qualtrics (35, 36).

### Measures

The outcome food insecurity was assessed using the United States Department of Agriculture six-item Food Security Survey Module (37). Participants were asked to answer the six-item module about the time period “Since the COVID-19 outbreak (March 1, 2020).” The six-item module was scored (range 0–6) consistent with USDA guidelines (37). Participants scoring 0–1 were classified as having high or marginal food security (food secure), and a score of 2–6 was classified as having low or very low food security (food insecure). The six-item Food Security Survey Module has a sensitivity of 92.0% and a specificity of 99.4% for overall food insecurity (38). Food-related worries were assessed by asking participants if they had worried about a set of eight food-related issues since the COVID-19 pandemic began (yes = 1, no or not applicable = 0), including food becoming more expensive, food becoming unsafe or contaminated, losing access to food assistance programs, not being able to afford enough food, not having enough food in stores, not having enough food stocks to stay home, the country not having enough food to feed everyone, and losing so much income that you cannot afford food. A food worry scale was computed (range 0–8), and high food-related worries were classified as worries above the mean (>4.65). Questions about food access were adopted directly from the NFACT survey, which achieved an alpha value of 0.70 (5). Negative impacts on employment during COVID-19 were used as a proxy for economic instability. Participants were asked if they experienced any employment changes since the pandemic began (lost job, reduced hours or income, furloughed, work from home, increased hours, no job changes, not applicable/do not work). Participants indicating job loss, reduced hours or income, or furlough were classified as having a negative job impact due to COVID-19 (negative job impact = 1, no negative job impact = 0).

Food assistance program use was assessed by asking participants to check all food assistance programs that their household had used since the pandemic began, including the Supplemental Nutrition Assistance Program (SNAP); Women, Infant, and Children's Program (WIC); school meal programs (including school lunch, breakfast, or summer meals), a food pantry or food bank; or other assistance programs such as the Commodity Supplemental Food program, Meals on Wheels, or other programs. A variable was created to measure the use of any food assistance program by classifying checking yes for any food assistance program (any/none).

To assess purchasing behavior, participants were asked whether they bought more, the same amount, or less of a set of goods, including fresh produce, frozen produce, snack foods, juice or soda, and frozen dinners. Five variables were created to capture increased purchasing since the pandemic (more = 1, the same or less = 0). The food source was assessed by asking participants where they purchased food since the pandemic began (grocery, convenience, or specialty store; grocery, meal kit, or Meals on Wheels delivery; restaurant take-out or dine-in; food pantry, school food, or meals served in a group setting; market of farmers, community-supported agriculture, or gardening). Five variables were created to capture food sources; if participants selected purchasing from any source within each category (store, delivery, restaurant, food assistance, or local), they were classified as purchasing food from that source.

Finally, demographic characteristics collected on the sample included income in 2019 (< $13,000, $13,000–24,999, $25,000–49,999, $50,000–74,999, $75,000+), gender (male, female, transgender, non-binary), education (high school or less, some college, 2-year degree, 4-year degree, graduate studies), and age (18–24, 25–34, 35–44, 45–54, 55–64, 65+).

### Data Analysis

Food security, food worries, food sources, food assistance use, purchasing behaviors, and demographics were described for the total sample. A chi-square analysis was performed to evaluate differences in the outcome food insecurity. To evaluate differences across data collection time points, food insecurity by race and ethnicity adjusting for the county of residence was examined in a logistic regression analysis. Using a model-building approach, factors independently associated with the outcome food insecurity were included in a set of logistic regression models (39). The first model examined individual worries related to food and negative job impacts, the second model added food sources, the third model added food assistance program use, the fourth model added purchasing behavior, and the final model added sample demographic characteristics. The log-likelihood, Akaike's information criterion (AIC), and Bayesian Information Criterion (BIC) were used to evaluate the model fit (39, 40). Analyses were performed in Stata version 16.1 (41).

## Results

The sample (*n* = 525) was 47% Black or African American, 42% Hispanic, and 11% non-Hispanic white and 62% female (Table 1). Over 30% of participants reported an income below $25,000 in 2019, a quarter of the sample (27%) reported an income between $25,000 and $50,000, 17% reported an income between $50,000 and $75,000, and the remaining 22% reported an income >$75,000. Approximately half of the sample reported less than a college degree with 28% reporting high school or less and 23% reporting some college but not graduating with a degree. Over 40% of participants were aged 18–34 years, about one-third of the sample was in the middle-age groups of 35–54, and the remaining quarter of respondents were aged 55 years and older.

**Table 1:** Demographic characteristics and food worries, food security, food assistance use, and purchasing behaviors of the sample, counts, and frequencies (*n* = 525).

	**Count**	**Percent**
Race/ethnicity		
Non-Hispanic White	60	10.8
Black or African American	260	46.9
Hispanic	234	42.2
Income		
< $13,000	100	18.1
$13,000–24,999	84	15.2
$25,000–49,999	150	27.1
$50,000–74.999	96	17.3
$75,000+	124	22.4
Gender		
Male	203	36.6
Female	346	62.5
Transgender	1	0.2
Non-binary	4	0.7
Education		
High school or less	155	28.0
Some college	130	23.5
2-year degree	104	18.8
4-year degree	119	21.5
Graduate studies	46	8.3
Age		
18–24	98	17.7
25–34	137	24.7
35–44	110	19.9
45–54	78	14.1
55–64	73	13.2
65+	58	10.5
High food worries	306	55.2
Food insecure since COVID-19	246	45.8
Negative job impact due to COVID-19	205	37.0
Food source: store	527	95.1
Food source: delivery	226	40.8
Food source: restaurant	406	73.3
Food source: food assistance	137	24.7
Food source: local food sources	180	32.5
Used any food assistance since COVID-19	273	49.3
Used SNAP since COVID-19	200	36.1
Used WIC since COVID-19	37	6.7
Used school meals since COVID-19	54	9.8
Used a food Bank since COVID-19	85	15.3
Used other food assistance since COVID-19	5	0.9
Purchased more fresh produce since COVID-19	209	37.7
Purchased more frozen produce since COVID-19	191	34.5
Purchased more snack foods since COVID-19	176	31.8
Purchased more juice/soda since COVID-19	155	28
Purchased more frozen dinners since COVID-19	150	27.1

Nearly half of the study participants (46%) reported experiencing food insecurity and 55% expressed a high level of food-related worries (Table 1). Over one-third of the sample (37%) reported a negative job impact during the COVID-19 pandemic. Most participants reported getting food from grocery stores (95%) and restaurants (73%). One-third of the participants (33%) reported using local food sources such as farmers' markets or community-supported agriculture, and 41% reported using delivery services to obtain food. One-quarter of the participants (25%) reported using food assistance programs for food supplies. Half of the study participants reported using any food assistance program (49%). More than one-third reported using SNAP (36%), 7% used WIC, 10% used a school meal program, and 15% used a food bank.

When looking at food purchasing (Table 1), 38% reported buying more fresh produce and 34% reported buying more frozen produce since the COVID-19 pandemic. When looking at purchasing less healthful foods, 32% of the participants reported buying more snack foods, 28% reported buying more sugar-sweetened beverages, and 27% reported buying more frozen dinners.

Bivariate analysis of high food-related worries, food sources, food assistance programs, and purchasing behavior by food security status since the COVID-19 pandemic showed that there were statistically significant differences between food-secure and food-insecure participants for high food-related worries, all food sources except for restaurants, all food assistance programs, and all purchasing behaviors (Table 2). Food-related worries were greater among participants reporting food insecurity (69%) than those reporting food security (44%). Negative job impacts were reported by a greater proportion of participants experiencing food insecurity since the pandemic (51%) compared to 25% of food-secure participants. Food-insecure participants reported using delivery (55%), food assistance (37%), and local food sources (40%) more frequently than food-secure participants (30, 14, and 26%, respectively). Participants experiencing food insecurity since the COVID-19 pandemic reported using all food assistance programs and purchasing more of all types of food more frequently than participants not experiencing food insecurity. The greatest difference in food assistance program use between the two groups was observed for the use of SNAP with 23% of food-secure participants and 51% of food-insecure participants reporting using the SNAP program (28-point difference). The greatest difference in food purchasing between the two groups was observed for frozen produce with 28% of food-secure participants and 43% of food-insecure participants reporting buying more frozen produce during the pandemic (15-point difference).

**Table 2:** Factors independently associated with food security since the pandemic, chi-square analysis (*n* = 525).

	**Food secure**		**Food insecure**		
	**Count**	**%**	**Count**	**%**	*p* diff^*^
High food worries	128	44.0	170	69.1	*p < * 0.001
Negative job impact due to COVID-19	73	25.1	127	51.6	*p < * 0.001
Food source: store	282	96.9	229	93.1	*p < * 0.05
Food source: delivery	89	30.6	135	54.9	*p < * 0.001
Food source: restaurant	210	72.2	186	75.6	0.366
Food source: food assistance	42	14.4	92	37.4	*p < * 0.001
Food source: local food source	77	26.5	99	40.2	*p < * 0.01
Used SNAP since COVID-19	67	23.0	126	51.2	*p < * 0.001
Used WIC since COVID-19	7	2.4	30	12.2	*p < * 0.001
Used school meals since COVID-19	13	4.5	41	16.7	*p < * 0.001
Used food bank since COVID-19	27	9.3	57	23.2	*p < * 0.001
Used other food assistance since COVID-19	3	1.0	2	0.8	0.793
Used any food assistance since COVID-19	95	32.7	171	69.5	*p < * 0.001
Purchased more fresh produce since COVID-19	94	32.3	111	45.1	*p < * 0.01
Purchased more frozen produce since COVID-19	81	27.8	7	43.5	*p < * 0.001
Purchased more snack foods since COVID-19	76	26.1	94	38.2	*p < * 0.01
Purchased more juice/soda since COVID-19	67	23.0	83	33.7	*p < * 0.01
Purchased more frozen dinners since COVID-19	62	21.3	85	34.6	*p < * 0.01
Race/ethnicity					
Non-Hispanic White	45	15.5	15	6.1	*p < * 0.001
Black or African American	146	50.2	104	42.3	
Hispanic	100	34.4	127	51.6	
Income					
< $13,000	43	14.8	49	19.9	*p < * 0.01
$13,000–24,999	36	12.4	46	18.7	
$25,000–49,999	72	24.7	74	30.1	
$50,000–74.999	59	10.3	36	14.6	
$75,000+	81	27.8	41	16.7	
Gender					
Male	115	39.5	84	34.2	0.199
Female, transgender, non-binary	176	60.5	162	65.9	
Education					
High school or less	76	26.1	71	28.9	*p < * 0.05
Some college	60	20.6	65	26.4	
2-year degree	48	16.5	53	21.5	
4-year degree	78	26.8	40	16.3	
Graduate studies	29	10.0	17	6.9	
Age					
18–24	39	13.4	54	22	*p < * 0.001
25–34	51	17.5	82	33.3	
35–44	62	21.3	45	18.3	
45–54	48	16.5	29	11.8	
55–64	44	15.1	26	10.6	
65+	47	16.2	10	4.1	

* *p difference from chi-square analysis*.

To evaluate the differences across data collection time points, the outcome food insecurity was examined by race and ethnicity adjusting for the county of residence as a proxy for disease burden variation across the two time points. For example, on August 15, 2020, there were zero cases in Delaware county and 50 cases in Erie county. On December 15, 2020, there were five cases in Delaware county and 396 cases in Erie county (34). Race and ethnicity were statistically significantly associated with the outcome food insecurity [odds ratio (OR) 1.88, 95% CI 1.44, 2.47], and the county was not significant in explaining variance in the outcome.

Factors independently associated with the outcome food insecurity were included in the multivariate analysis (Table 3). In model one, food-related worries and negative job impact due to COVID-19 were examined with the outcome food insecurity. Participants with high food-related worries were more than two and a half times more likely to report food insecurity since the pandemic (OR 2.60, 95% CI 1.80, 3.75). Participants reporting a negative job impact due to COVID-19 were nearly three times more likely to report food insecurity since the pandemic compared to participants with no impact or an increase in income or hours (OR 2.92, CI 2.01, 4.25).

**Table 3:** Factors associated with food security since the pandemic, logistic regression (*n* = 525).

	**Model 1**		**Model 2**		**Model 3**		**Model 4**		**Model 5**	
	OR^*^	**95% CI**	**OR**	**95% CI**	**OR**	**95% CI**	**OR**	**95% CI**	**OR**	**95% CI**
High food worries	**2.60**	**1.80, 3.75**	**3.21**	**2.14, 4.82**	**2.74**	**1.81, 4.16**	**2.58**	**1.69, 3.96**	**2.82**	**1.81, 4.40**
Negative job impact due to COVID-19	**2.92**	**2.01, 4.25**	**2.34**	**1.57, 3.48**	**2.41**	**1.58, 3.66**	**2.34**	**1.53, 3.56**	**2.20**	**1.42, 3.42**
Food source: store			**0.38**	**0.15, 0.97**	0.39	0.15, 1.03	0.39	0.15, 1.04		
Food source: delivery			**2.22**	**1.49, 3.30**	**2.35**	**1.54, 3.59**	**2.21**	**1.43, 3.40**	**2.31**	**1.46, 3.65**
Food source: food assistance			**2.59**	**1.62, 4.16**	**2.06**	**1.22, 3.48**	**1.97**	**1.16, 3.34**	**1.97**	**1.16, 3.34**
Food source: local food source			1.53	0.99, 2.39						
Used SNAP since COVID-19					**3.19**	**2.09, 4.88**	**3.19**	**2.08, 4.90**	**3.21**	**1.98, 5.21**
Used WIC since COVID-19					**3.11**	**1.21, 7.99**	**3.16**	**1.22, 8.14**	2.39	0.89, 6.39
Used school meals since COVID-19					2.00	0.94, 4.27	2.01	0.93, 4.32		
Used food bank since COVID-19					1.33	0.72, 2.46	1.35	0.73, 2.50		
Purchased more fresh produce since COVID-19							1.17	0.76, 1.83		
Purchased more frozen produce since COVID-19							1.31	0.83, 2.07		
Purchased more snack foods since COVID-19							1.09	0.66, 1.78		
Purchased more juice/soda since COVID-19							0.96	0.58, 1.58		
Purchased more frozen dinners since COVID-19							1.01	0.60, 1.70		
Race/ethnicity										
Non-Hispanic White									ref	
Black or African American									1.26	0.57, 2.80
Hispanic									**2.40**	**1.07, 5.40**
Income										
< $13,000									ref	
$13,000–24,999									1.29	0.62, 2.69
$25,000–49,999									1.12	0.58, 2.16
$50,000–74.999									0.65	0.31, 1.37
$75,000+									0.71	0.33, 1.52
Education										
High school or less									ref	
Some college									1.16	0.64, 2.12
2-year degree									1.26	0.65, 2.44
4-year degree									0.77	0.40, 1.49
Graduate studies									1.21	0.49, 2.98
Age										
18–24									ref	
25–34									0.74	0.39, 1.42
35–44									0.52	0.26, 1.02
45–54									**0.45**	**0.21, 0.97**
55–64									0.55	0.25, 1.19
65+									**0.35**	**0.14, 0.91**
Log likelihood	−336.76		−308.34		−287.40		−285.97		−274.38	
AIC	679.53		630.67		594.80		601.94		594.75	
BIC	692.39		660.67		637.66		666.23		693.33	

* *Odds ratios and 95% confidence intervals reported from the logistic regression analysis.*

*Bolded numbers indicate a statistically significant finding*.

In model two (Table 3), food sources were examined. High food-related worries (OR 3.21, CI 2.14, 4.82), negative job impact due to COVID-19 (OR 2.34, CI 1.57, 3.48), using delivery (OR 2.22, CI 1.49, 3.30), and using food assistance (OR 2.59, CI 1.62, 4.16) increased the likelihood of food insecurity, and getting food from grocery stores (OR 0.38, CI 0.15, 0.97) decreased the likelihood of food insecurity. In post-testing, the use of local foods did not strengthen the model and therefore, was dropped from the subsequent analysis.

In model three (Table 3), food assistance programs were added. High food-related worries (OR 2.74, CI 1.81, 4.16), negative job impact (OR 2.41, CI 1.58, 3.66), use of delivery (OR 2.35, CI 1.54, 3.59), and use of food assistance (OR 2.06, CI 1.22, 3.48) persist in explaining the variance in the outcome food insecurity and grocery store use is explained away with the addition of food assistance measures. SNAP (OR 3.19, CI 2.09, 4.88) and WIC use (OR 3.11, CI 1.21, 7.99) increased the odds of food insecurity.

In model four (Table 3), purchasing behaviors were added to the model. Purchasing behaviors were not statistically significant in explaining the variance in the outcome food security. Food-related worries (OR 2.58, CI 1.69, 3.96), job disruption (OR 2.34, CI 1.53, 3.56), delivery (OR 2.21, CI 1.43, 3.40), food assistance use (OR 1.97, CI 1.16, 3.34), SNAP (OR 3.19, CI 2.08, 4.90), and WIC use (OR 3.16, CI 1.22, 8.14) persist as accounting for the variance in the outcome. In post-testing, the use of school meals and food banks and purchasing behaviors did not strengthen the model. These measures were dropped from the subsequent modeling.

The final model (Table 3) added sample demographic characteristics. After accounting for sample characteristics, high food-related worries, job disruption due to COVID-19, food assistance, SNAP use, and Hispanic ethnicity were associated with greater odds of food insecurity since the COVID-19 pandemic and older age was associated with decreased odds of food insecurity. Participants with a high level or food-related worries were 2.82 times more likely (OR 2.82, CI 1.81, 4.40) to report food insecurity since the pandemic. Participants reporting a negative job impact during the pandemic were more than twice as likely to report food insecurity than participants without a job disruption or an increase in hours or income (OR 2.20, CI 1.42, 3.42). Participants reporting getting food through delivery services were more than twice as likely (OR 2.31, CI 1.46, 3.65) to report food insecurity. SNAP users in the sample were more than three times more likely (OR 3.21, CI 1.98, 5.21) to report food insecurity compared to non-SNAP users. Hispanic participants were nearly two and a half times more likely (OR 2.40, CI 1.07, 5.40) to report food insecurity compared to non-Hispanic white participants. Participants aged 65 or older were 65% less likely (OR 0.35, CI 0.14, 0.91) to report food insecurity compared to younger participants (aged 18–24 years). Evaluation of AIC and BIC indicated that with the addition of each set of food-related variables, model fit improved with the exception of model 4 (40, 42).

## Discussion

This study examined factors associated with food insecurity among New York State residents that oversampled for Black, Hispanic, and low-income and education. After accounting for other factors, higher food worries, a negative job impact, SNAP use, self-reported Hispanic ethnicity, and reported food assistance and delivery as food sources were associated with a higher likelihood of experiencing food insecurity.

Research on food insecurity during COVID-19 at the national and local levels and among high-risk populations, such as low-income, low education, and Black, Indigenous, and people of color (BIPOC), are emerging throughout the United States. Studies have commonly reported or projected increased rates of food insecurity during COVID-19 (4–7, 43–47). National studies examining food insecurity, challenges, and worry have reported increased rates of food insecurity among low-income households (43, 47), households with low education (6, 43), BIPOC households (6, 43, 44, 47), households with children (6, 44, 47), those experiencing employment disruption (6, 43, 44), and SNAP participants (6). A nationally representative study on low-income adults found that they were less able to comply with recommendations to stock up on 2 weeks of groceries and more likely to need extra money for food and bills (6). Food-insecure households experienced more fear and worry about COVID-19 in general (44) and about food during COVID-19 in particular (45).

Similar findings were reported in studies in specific states or localities. A study of emerging adults in Minnesota found that food-insecure adults reported eating less and expressed food-related concerns, such as worry about the safety of going to stores (46). A longitudinal study in Pittsburgh in two low-income, predominantly African American neighborhoods found that COVID-19 led to increased rates of food insecurity (48). Food bank and SNAP participation remained unchanged in these communities during the pandemic, suggesting that the existing safety nets were failing to reach those with emerging needs. Dubowitz et al. (48) also found that psychological distress, loss of work, and concerns about leaving home to buy food contributed to food insecurity. Lastly, a study in Vermont found higher rates of food insecurity among respondents experiencing job disruption, households with children, and among respondents with low income and education (5). Food-insecure respondents were more likely to experience challenges with food access and to express higher food-related worry (5). Although, national and local studies conducted to date on food access and insecurity during COVID-19 report similar findings, the variation in state response to the pandemic (i.e., stay at home order timing and length and food assistance program waiver requests) and projected impacts of the pandemic on food insecurity by state and locality (7) warrant examinations of food insecurity and food access challenges and worry at the state and local levels.

The results of this study align with much of the work that has been conducted on food access and insecurity during COVID-19. Participants indicating a high level of pandemic food-related worries were more likely to be food-insecure since the pandemic. The high level of pandemic food worries among food-insecure study participants may be related to the high level of economic, health, and social disruption created by the pandemic (12, 14). Food supply fluctuations created by the COVID-19 pandemic may be contributing to worries about there being enough food in the food supply or securing sufficient food supplies for their household (4, 5, 8). Hispanic participants had an increased likelihood of food insecurity compared to non-Hispanic white participants in this sample. This aligns with the greater burden of food insecurity experienced by Hispanic-headed households in non-pandemic times with a prevalence of 15.6% in 2019 compared to 7.9% among non-Hispanic white households (23). It also aligns with a greater burden of COVID-19 impacts falling on minority individuals (17, 19, 49). Job loss or disruption since the pandemic was associated with higher odds of experiencing food insecurity. The pandemic resulted in the loss or disruption of millions of jobs; in the United States, the unemployment rate increased from 3.5% in February 2020 to a high of 14.8% in April 2020, marking the highest unemployment rate since the Current Population Survey began collecting data (50). Loss of income has been linked to food insecurity both before and since the pandemic (5, 51).

Supplemental Nutrition Assistance Program use was associated with an increased likelihood of food insecurity. The SNAP program was designed to increase access to healthy food for eligible low-income households and consequently improve food security (52, 53). There are documented challenges with measuring food insecurity among SNAP participants due to the self-selection bias—people that are food-insecure are more likely to seek food assistance (53). However, studies of food insecurity among SNAP users accounting for the self-selection effect have found a reduction in food insecurity among new SNAP participants that persists into program usage (53–55). Given the documented food insecurity prevalence among SNAP users, we posit that the increased likelihood of food insecurity may be related to unmet needs during the pandemic among study participants or due to greater anxiety or concern related to uncertainty and the economic and health impacts of the pandemic. If the association between SNAP use is related to unmet needs, this may highlight the importance of greater funding levels for SNAP participants. Additional research on food insecurity among SNAP users during the pandemic with monthly data would shed additional light on the food insecurity burden among SNAP participants.

No studies have reported on the relationship between food purchasing behaviors and food insecurity during COVID-19. The only purchasing behaviors that were significant in this analysis were reported food sources. The rationale behind including specific food types (healthy and less healthy) was to examine if participants purchasing less healthy foods were more likely to experience food insecurity, and it appears that food source (food assistance use and delivery) is more indicative of food insecurity than purchasing any specific food category. In developing new interventions for supporting families and food access, food delivery and food assistance programs may be useful avenues for increasing food distribution or outreach to target populations. Further, amid uncertainty and food chain disruptions, emphasizing healthy food availability through these channels may better support food-insecure families than other outlets.

This study has several limitations to bear in mind when considering the results. The study design was cross-sectional; therefore, analyses are not able to determine causal relationships. To mitigate this limitation, survey questions asked respondents specifically about purchasing behavior, food sources, food worries, and food assistance in relation to COVID-19. The purposive quota sampling frame intentionally oversampled minority and low-income New Yorkers to recruit a sufficient sample size of groups with an increased risk for COVID-19 infection and complications, as well as with an increased risk for food insecurity. With COVID-19 disparities, understanding the experiences of individuals with greater risk was prioritized over representativeness. As a result of purposive quota sampling, generalizations cannot be made about New Yorkers. Data on non-Hispanic white participants were collected ~3 months after data on Black and Hispanic participants. The economic impacts of the pandemic were ongoing during this time; enhanced unemployment insurance benefits had expired and a second economic relief bill had not yet been passed. Further, with a greater disease burden in December compared to the summer months when data were collected from Black and Hispanic participants, we would expect disparities to be underestimated rather than overestimated. Regional variation in case counts, which remained over time, posed an additional limitation on the analysis. County residence as a proxy for different disease burden between time points adjusted for race and ethnicity was not significant in explaining the variance in the outcome. The survey was administered through a web-based platform; therefore, only individuals with internet access were able to participate in the study. Most Americans (89% overall, 88% of Hispanics, and 87% of Black Americans) have access to the internet; however with economic disruption, there may be increased accessibility challenges (56). Given the widespread impact of the pandemic in New York State, a web survey was selected to facilitate timely completion of data collection and provides an important snapshot of the lived experiences of many New Yorkers during the pandemic.

Future research are needed to assess the ongoing impacts of the pandemic on food access and food insecurity, particularly among underserved groups. Concerns about food during this time may be important factors related to reduced food security and policy efforts to address these concerns may be necessary. Measures that provide additional money for food, support price reductions for food delivery, and support the provision of healthy foods through food assistance programs may alleviate barriers to accessing enough healthy food. Making current expansions in food assistance program benefits permanent may improve food access as the United States recovers from COVID-19 and in the long term, as we know that food assistance take-up rates decline as benefits decline (57). Focusing on food assistance, however, may not be enough. The causes of food insecurity extend beyond low-income to include physical and mental health, family structure, and access to child care, to name a few, so policy efforts that expand access to health care and child care may provide additional support for families to meet their basic needs, including food. In particular, policies that streamline the process to apply for SNAP benefits across states, improve access to mental health services, incentivize or require the adoption of Medicaid expansion, and increase funding for child care may help families access food in the future and during times of crisis.

## Data Availability Statement

The raw data supporting the conclusions of this article will be made available by the authors, without undue reservation.

## Ethics Statement

The studies involving human participants were reviewed and approved by D'Youville College IRB. The patients/participants provided their Written Informed Consent To Participate In This Study.

## Author Contributions

LC was involved in conceptualization, methodology, and funding acquisition. LC and SR were involved in analysis, writing the original draft preparation, and writing the review and editing. Both authors have read and agreed to the published version of the manuscript.

## Conflict of Interest

The authors declare that the research was conducted in the absence of any commercial or financial relationships that could be construed as a potential conflict of interest.

## Publisher's Note

All claims expressed in this article are solely those of the authors and do not necessarily represent those of their affiliated organizations, or those of the publisher, the editors and the reviewers. Any product that may be evaluated in this article, or claim that may be made by its manufacturer, is not guaranteed or endorsed by the publisher.
